# SPOCK1 overexpression suggests poor prognosis in head and neck squamous cell carcinomas

**DOI:** 10.3389/pore.2026.1612448

**Published:** 2026-08-06

**Authors:** Noemi Piroska Jakob, Matyas Majoros, Imre Uri, Helga Szakadati, Ede Birtalan, Kornelia Baghy, Zoltan Kelemen, Laszlo Tamas, Kornel Danos, Tibor Krenacs

**Affiliations:** 1 Department of Pathology and Experimental Cancer Research, Semmelweis University, Budapest, Hungary; 2 Department of Otorhinolaryngology, Head and Neck Surgery, Semmelweis University, Budapest, Hungary; 3 Department of Oro-Maxillofacial Surgery and Stomatology, Faculty of Dentistry, Semmelweis University, Budapest, Hungary

**Keywords:** head and neck squamous cell carcinoma, immunohistochemistry, mRNA expression, prognostic biomarker, SPOCK1 proteoglycan

## Abstract

Head and neck squamous cell carcinomas (HNSCCs) are mostly associated with smoking, alcohol intake, and/or human papillomavirus HPV16 and/or HPV18 infection. However, most tumors are diagnosed at an advanced stage. In the search for biomarkers of HNSCC, we studied the prognostic role of SPOCK1, a heparan sulfate proteoglycan. In HNSCC, SPOCK1 expression was tested both at *in silico* mRNA level using the Kaplan-Meier Plotter and at *in situ* protein level after SPOCK1 immunohistochemistry after whole slide digitalization. Protein expression results were analyzed using H-Scores, both by eye-control testing and with a machine-learning-based automated image analysis tool, DensitoQuant. SPOCK1 mRNA expression was found to be significantly higher in oral carcinomas than in the normal oral mucosa (log-rank p = 5.76e-06). Elevated SPOCK1 mRNA expression was consistently associated with a strongly reduced overall survival (OS; HR = 1.57 (1.2–2.06); log-rank p = 0.001) in HNSCC. This was particularly true for tumors with high (log-rank p = 0.00085) vs. low (log-rank p = 0.55) neoantigen (NeoAG) levels, at median antigen level stratification. Both the Cox-regression analysis and the log-rank test revealed elevated SPOCK1 protein levels in association with significantly reduced OS in our 97 HNSCC patients (both p = 0.002), which was supported by automated image analysis testing (p = 0.008 and p = 0.044, respectively). Our findings suggest that SPOCK1 can be a candidate adverse prognostic biomarker also in HNSCC. Therefore, SPOCK1 overexpression may specify a group of oral cancers with aggressive tumor biology.

## Introduction

Head and neck squamous cell carcinomas (HNSCCs) originate from the mucosal lining of the oral cavity, pharynx, and larynx, making them the most prevalent cancers in this anatomical region [1]. According to the GLOBOCAN cancer estimates ∼890,000 new HNSCC cases were diagnosed in 2018, resulting in ∼450,000 annual deaths, which account for 4.5% of all cancer mortality [2]. The incidence of HNSCC varies globally and is strongly linked to risk factors such as tobacco use and excessive alcohol intake as well as [2] human papillomavirus (HPV) strains, particularly HPV-16 and, to a lesser extent, HPV-18 [3, 4]. Since these high-risk HPV strains are included in FDA-approved vaccines, widespread immunization efforts can potentially prevent HPV-positive HNSCC [1]. Oral cancers related to other causes, particularly smoking are classified as HPV-negative HNSCC [5]. Although some oral precancerous lesions of squamous cell carcinoma, such as leukoplakia (white patches) and erythroplakia (red patches), may progress into invasive disease, most patients are diagnosed at an advanced stage [1]. Since the molecular knowledge behind HNSCC development and progression is still incomplete, searching for novel molecular biomarkers associated with tumor behavior is still relevant.

In HNSCC, cumulative genetic instability drives epithelial stem cells through the hyperplasia, dysplasia, carcinoma *in situ* and invasive carcinoma progression sequence [1, 6, 7]. This involves e.g., the loss of regions of 9p21 encoding for the tumor suppressor CDKN2A (p16INK4A a CDK4 and CDK6 inhibitor) and ARF (p14, a stabilizer of p53 protein) besides the progressive loss of different parts of the TP53 gene on chromosome 17. Accordingly, several stem cell biomarkers have been detected in HNSCC, including CD44 [8], ALDH1 [9, 10], CD133, SOX2, OCT4, KLF4 and NANOG [11–13].

Extracellular matrix (ECM) and stromal components, have only been recently recognised as important contributors of cancer initiation and progression of malignant tumors [14–20]. SPOCK1 (testican-1), first identified in human seminal plasma [21], is a heparan sulfate/chondroitin sulfate proteoglycan [22]. The SPOCK family members (SPOCK1, SPOCK2, and SPOCK3) are highly conserved ECM proteoglycans with multiple functional domains [23]. When overexpressed, SPOCK may promote cell cycle progression and tumor cell growth by upregulating the PI3K/AKT signaling e.g., in breast, gallbladder, ovarian, colorectal and liver cancers [24–27]. It may also be involved in the elevated invasion and migration e.g., of gastric cancer, glioma, as well as in pancreatic and prostate cancer [17, 19, 28, 29]. Recent studies highlighted the role of SPOCK1 in the activation of cell migration [30, 31] and matrix metalloproteinases (MMPs, e.g., MMP-2), which can support epithelial-to-mesenchymal transition (EMT) in cancer progression [32]. Our group has recently shown in mouse, rat and human liver models that in hepatocarcinogenesis SPOCK1 is upregulated in transformed hepatocytes, with high levels observed in hepatocellular carcinomas and cirrhotic liver, which latter may transform into cancer, but not in the extracellular matrix of the liver [24]. Also, we detected SPOCK1 upregulation in ovarian cancer in relation to poor disease outcome [33].

In the oral cavity of transgenic mice, elevated SPOCK1 expression mediated EMT in a cyclosporin-A induced gingival overgrowth model [34, 35]. Oral mucosa showed an elevated probability for EMT due to increased secretion of MMP9 and reduced TNFα and IL-1β levels. EMT featured by reduced E-Cadherin and emerging N-Cadherin and vimentin production may also be involved in HNSCC progression resulting in increased motility, stromal spreading, stemness and therapy resistance [36, 37]. These observations suggested that SPOCK1 may also influence the basic functions and potentially the malignant transformation and progression of oral epithelial tissues.

In this study we investigated the prognostic role of SPOCK1 expression in HNSCC by using both the *in silico* mRNA analysis tool, Kaplan-Meier Plotter and the *in situ* protein expression of different oral cavity locations applying standardized immunohistochemistry. H-Score results were gained in digital sections both by eye-control testing and using a machine-learning-based automated image analysis tool called DensitoQuant.

## Materials and methods

### *In silico* mRNA analysis

First, we tested the prognostic value of SPOCK1 mRNA expression using an open-source database management software package, Kaplan–Meier Plotter [38, 39] (http://kmplot.com). Overall survival (OS) data of 500 HNSCC patients obtained from the Gene Expression Omnibus, GEO database were extracted through the „Pan-Cancer” algorithm of the program. Patients were stratified into high and low expressors by dichotomizing at their quartiles or median, and the patients who survived the given period of the analysis were censored. Some analyses were also restricted to tumor subtypes or immune cell content.

### Patient and sample selection

For the immunohistochemical study tissue samples routinely fixed in 10% formalin and embedded in paraffin were obtained from 97 patients (79 men, 18 women) diagnosed with and treated for HNSCC at the Departments of Pathology, as well as Otorhinolaryngology and Head and Neck Surgery of Semmelweis University between 2012 and 2015. All tissue samples were handled in a coded fashion, according to the Dutch and Hungarian National Ethical guidelines (National Ethical Review Board approval: TUKEB, under the BM/17538-1/2024 number). The Committee waived the need for individual patient consent on archived tissues available for primary diagnostics for the purpose of retrospective biomarker testing.

The most common anatomical location of HNSCC studied was oropharyngeal tumors (n = 36; 37.1%). Within this group, 12 patients had p16-positive, 23 had p16-negative, and one had unknown p16 status. The main features of the studied HNSCC cases are summarized in Table 1.

**TABLE 1:** Features of the tested HNSCC cases.

​	Oral cavity	p16 pos. Oropharynx	p16 neg. Oropharynx	Oropharynx, p16 unknown	Hypopharynx	Supraglottic larynx	Glottic larynx	Subglottic larynx
Total count	2	12	23	1	22	11	25	1
Percent of total	2.1	12.4	23.7	1.0	22.7	11.3	25.8	1.0
Sex								
Male	1	9	15	1	21	8	23	1
Female	1	3	8	0	1	3	2	0
Age (mean)	61	57	62	50	60	58	61	69
Stage								
1	1	0	1	0	0	1	8	0
2	0	5	2	0	0	3	7	0
3	0	5	2	0	4	3	5	0
4	1	0	13	0	17	3	2	1
Missing	0	2	5	1	1	1	3	0
ECOG								
0	​	8	16	1	12	7	15	​
1	1	3	4	​	8	4	7	1
2	1	1	3	​	1	0	2	​
3	​	0	0	​	1	0	1	​
Five year survival								
No	1	8	17	0	17	3	10	0
Yes	1	4	6	1	5	8	15	1

Histopathological re-examination confirmed squamous cell carcinoma in all cases. Overall survival in the population was 59.2 ± 53.6 months (range: 0.7–154.9). A minimum 5-year survival was observed in 42.3% of patients (41 individuals). The average age of the patients was 60.3 ± 8.6 years (range: 41.2–78.9). The average BMI was 24.2 ± 4.9 kg/m^2^ (range: 15.5–37.7), and the average weight loss was 5.1%. General health status was ECOG grade 0 in 60.8% of cases (59 patients). Among comorbidities, diabetes mellitus was present in 11 patients, and ischemic heart disease in 38 patients. Smoking and alcohol consumption were the most common risk factors. Currently, 60% of the participants (58 individuals) are smokers, and 38 (39.2%) are regular alcohol consumers.

### Tissue microarrays and immunohistochemical analysis

After selecting representative tumor areas on the hematoxylin-eosin-stained slides, tissue microarrays (TMA) were created using a computer-driven semi-automated instrument (TMAMaster, 3D HISTECH Ltd., Budapest, Hungary). Duplicate or triplicate cores of 2 mm diameter were arrayed from the tumor samples into the recipient blocks. Altogether, 5 TMA blocks were created.

The TMA blocks were cut into 3 µm thick sections onto TOMO adhesive microscope slides (Matsunami Co, Osaka, Japan). After routine dehydration in a series of alcohol and xylene, antigen retrieval was performed using an electric pressure cooker (Avair, Biofa, Veszprem, Hungary) in Tris-EDTA buffer (pH 9.0, 0.1 M Tris base, 0.1 M EDTA) for 20 min. Following incubation of the TMA sections with monoclonal mouse anti-SPOCK1 antibody (Sigma, HPA007450) at a 1:100 dilution at 4 °C overnight, the Histols-MR-T (Histopathology Ltd., Pécs, Hungary) polymer peroxidase detection system was used for 30 min, at room temperature, and the reaction revealed in brown, using a H_2_O_2_/DAB substrate chromogen system. Liver sections were used as external positive and negative controls (without using primary antibody). Sections were washed between incubation steps 3 × 5 min in a 0.01 M Tris-buffered saline (TRIS) buffer (pH 7.4), counterstained using hematoxylin, and coverslip-mounted after dehydration. Finally, all TMA slides were digitized using a Panoramic scan instrument (3D HISTECH) equipped with a ×20 Carl Zeiss objective (NA = 0.83; Carl Zeiss MicroImaging Inc., Jena, Germany).

Digitalized whole TMA slides were examined with the Pannoramic Viewer program either by eye-control and/or using the DensitoQuant algorithm of the QuantCenter program package under the same concept, both considering the staining intensity and the proportion of positive cells. Immunostained tumors were classified into negative (0), mild (1), moderate/medium (2) and strong (3) intensity groups either using eye-control or by setting up threshold values for automated evaluation. These scores were multiplied by the percentage of tumor cells showing the given reaction intensity, resulting in a sum score (H-Score). Therefore, whether established by eye or by the software, the H-Score could be between 0–300. Due to some tumor heterogeneity, the visual determination of the proportional intensity scores within annotations might carry more bias than by DensitoQuant, an image-segmentation-based algorithm, which automatically highlighted the different intensities of each pixel and the determined exact area. At least 10 threshold pre-settings were tested before the final setting was uniformly used to all samples for annotated tumor areas. four-tier (0–3) intensity categories were measured in %, making up an H-Score for each 3-5 representative areas on each sample. Scoring by three independent assessors (MM, NJ and TK) using eye-control was done on the same 3 annotations per sample by both approaches. The final, eye-controlled results, were consolidated upon agreement of the assessors.

### Statistical analysis

The statistical processing and evaluation of the data were performed using the Spotfire Statistica software (version 14.2.0). For survival parameter testing, Cox-regression analysis, Chi-square tests of H-Score quartiles and log-rank tests were applied. In the latter, patients were divided into “high” and “low” expressors at their upper and lower quartiles or median of SPOCK1 expression levels. The weighted number of events (WW) was also determined using the Log-rank tests, and visual analysis of Kaplan-Meier survival curves was drawn. The survival difference of high and low expressors was considered significant at p < 0.05.

To detect more subtle differences, Cox regression was preferred for assessing the strength of the effect of the examined parameter on survival (RR, relative risk), compared to the Log-rank test. Chi-square test of H-Score quartiles, Log-rank tests and Cox regressions were performed. The results showing significant associations were highlighted.

## Results

### SPOCK1 mRNA expression-related survival in head and neck squamous cell carcinoma

**FIGURE 1:**
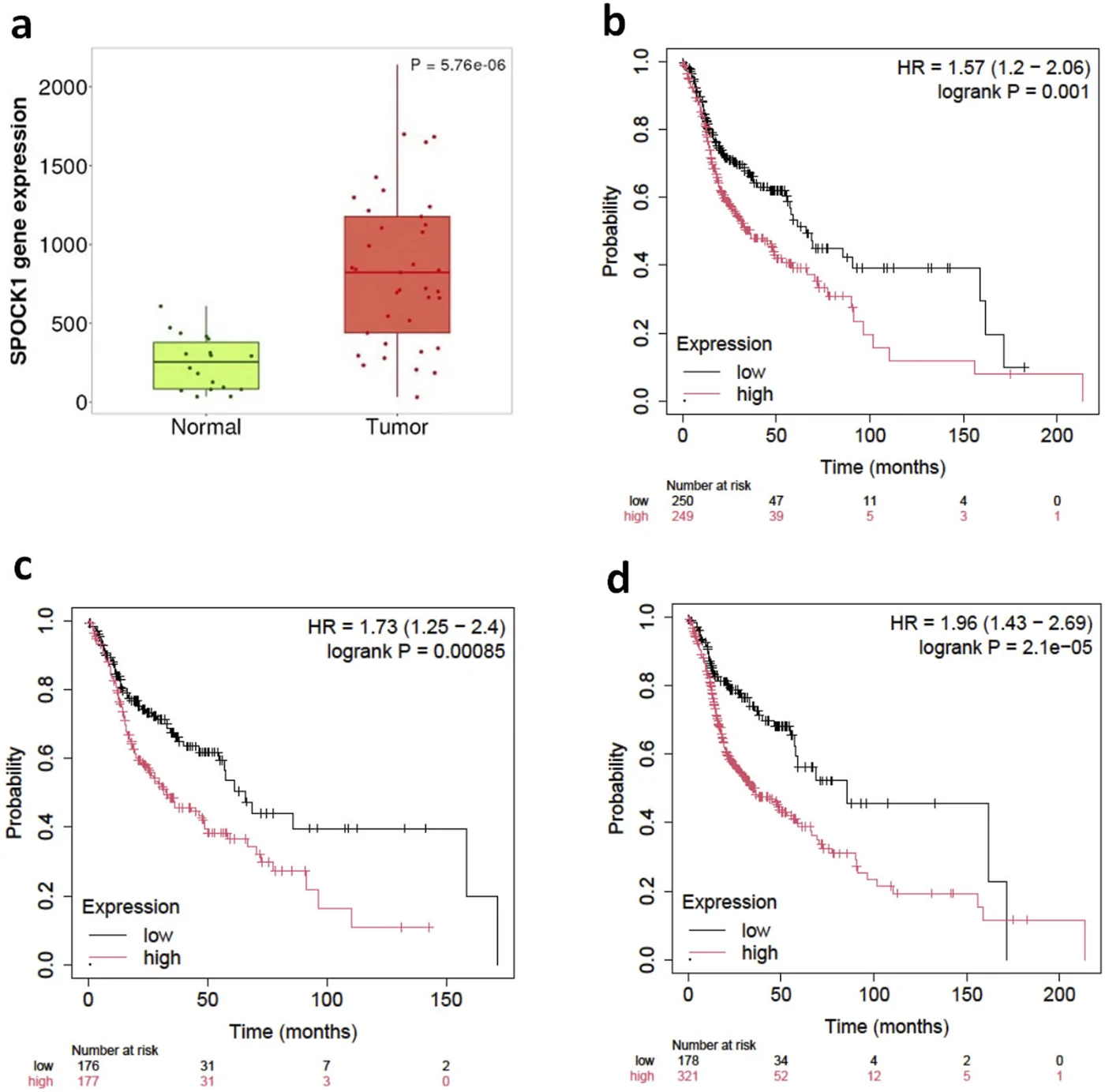
Analysis of SPOCK1 mRNA expression in normal oral mucosa, oral cancer and head and neck squamous cell carcinoma (HNSCC). Significantly higher SPOCK1 mRNA levels were detected in cancer of the oral cavity than in the normal oral mucosa **(a)**. KM-Plotter data show significantly worse overall survival (OS) of HNSCC patients with elevated SPOCK1 expression **(b)**, especially in cases with high neoantigen (NeoAG-high) levels **(c)**, when both data were split at median expression. The significance of SPOCK1 mRNA expression for adverse OS was further increased when SPOCK1 expression was dichotomized at the best expression cut-off **(d)**.

Analysis of publicly available transcriptomic datasets of 500 HNSCC patients using the Pan-Cancer algorithm of the KM-Plotter database demonstrated that SPOCK1 mRNA expression was markedly higher in carcinomas than in the normal oral mucosa (log-rank p = 5.76e-06) (Figure 1a). In HNSCC, elevated SPOCK1 expression was consistently associated with significantly reduced OS (log-rank p = 0.001, HR = 1.57 (1.2–2.06) (Figure 1b). This was particularly true in tumors with high (log-rank p = 0.00085, HR = 1.73 (1.25–2.4) (Figure 1c) vs. low (log-rank p = 0.55, HR = 1.18 (0.68–2.07) neoantigen (NeoAG) levels, at median SPOCK1 expression. The adverse role of SPOCK1 mRNA expression further increased when patients were stratified at the optimal prognostic cut-off (log-rank p = 2.1e-05, HR = 1.96 (1.43–2.69) (Figure 1d). These data suggested that SPOCK1 mRNA overexpression in HNSCC can be a clinically meaningful indicator of an aggressive tumor behavior.

### SPOCK1 protein expression-related survival in head and neck squamous cell carcinoma

#### Eye-control based H-Score related HNSCC survival

**FIGURE 2:**
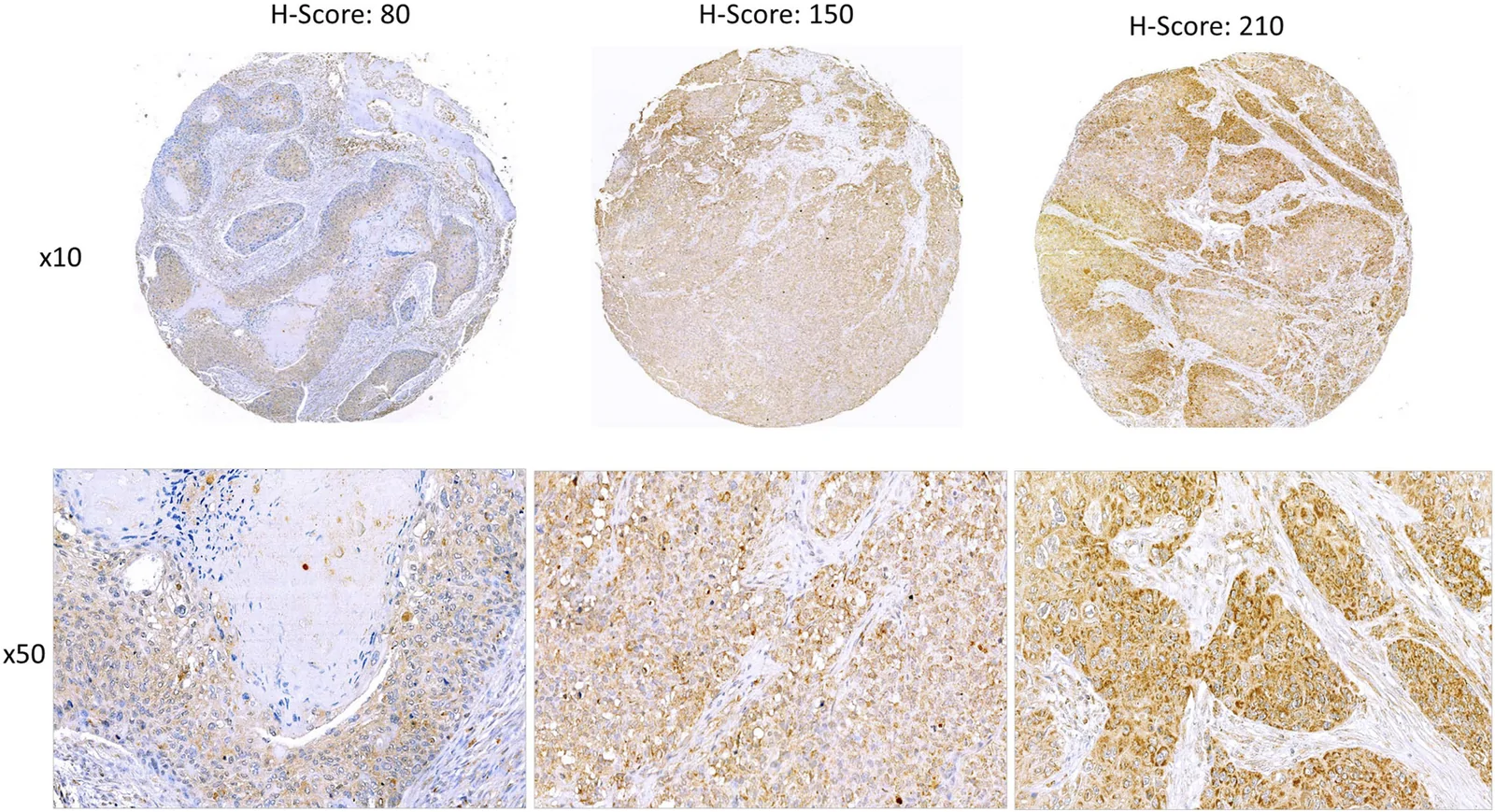
Examples of H-Score values given by eye-control testing for SPOCK1 protein expression detected using immunohistochemistry. The scale bar represents: 500 µm on the upper and 100 µm on lower image series.

SPOCK1 protein expression in HNSCC tested by eye-control on immunostained digital slides was semiquantitatively scored on the same annotations as those used later for automated analysis according to the H-score system, which considers both the intensity (0–3) and the percentage of positive tumor area. These values were then multiplied by each other. The granular peri-, and paranuclear immunohistochemical reactions suggested that the SPOCK1 protein mainly localized to the Golgi-region, the mitochondria, and rarely in the nucleus of tumor cells, practically without any stromal positivity. Examples of representative tumor regions demonstrated low (H-score 80), intermediate (H-score 150), and high (H-score 210) protein expression across HNSCC cases (Figure 2).

**FIGURE 3:**
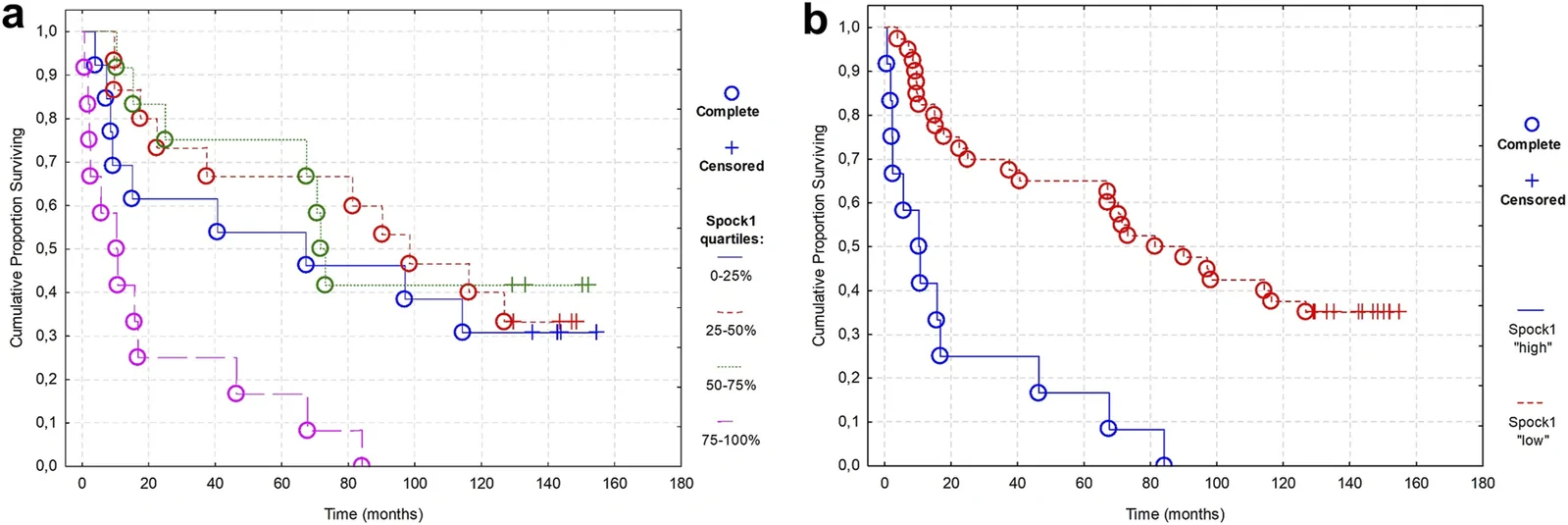
Overall survival (OS) of HNSCC patients based on SPOCK1 immunohistochemistry using H-Score results (average intensity multiplied by positive tumor area). Elevated SPOCK1 protein expression shows significant association with reduced overall (OS) survival using the Chi-square test of H-Score quartiles [p = 0.004, χ2 = 13.202, df = 3; **(a)**]. Log-rank test also demonstrates that patients with elevated SPOCK1 levels had worse OS, when H-Scores were split at the upper quartile [p = 0.002, WW = 8.003, Test statistic = 3.105; **(b)**].

Eye-control based H-Score results were plotted against the survival of HNSCC patients using a Kaplan-Meier survival curve. Cox regression based on visual scoring demonstrated a significant association between SPOCK1 expression and overall survival (p = 0.002, χ2 = 9.753, df = 1) with relative risk (RR) of 1.017 (95% CI: 1.006–1.027). Chi-square test of the H-Score quartiles revealed that elevated SPOCK1 protein levels were associated with significantly poorer OS (p = 0.004, *χ* ^*2*^ = 13.202, df = 3) (Figure 3a). The Log-rank test also showed significant detriment of OS, when all data were split at the upper quartile of H-Scores (p = 0.002, WW = 8.003, test statistic = 3.105) (Figure 3b), but not when split at the median or the lower. All in all, these results are in line with mRNA expression data and further support the role of SPOCK1 as a clinically relevant negative prognostic biomarker in HNSCC.

#### Digital image analysis-based H-Score related HNSCC survival results

**FIGURE 4:**
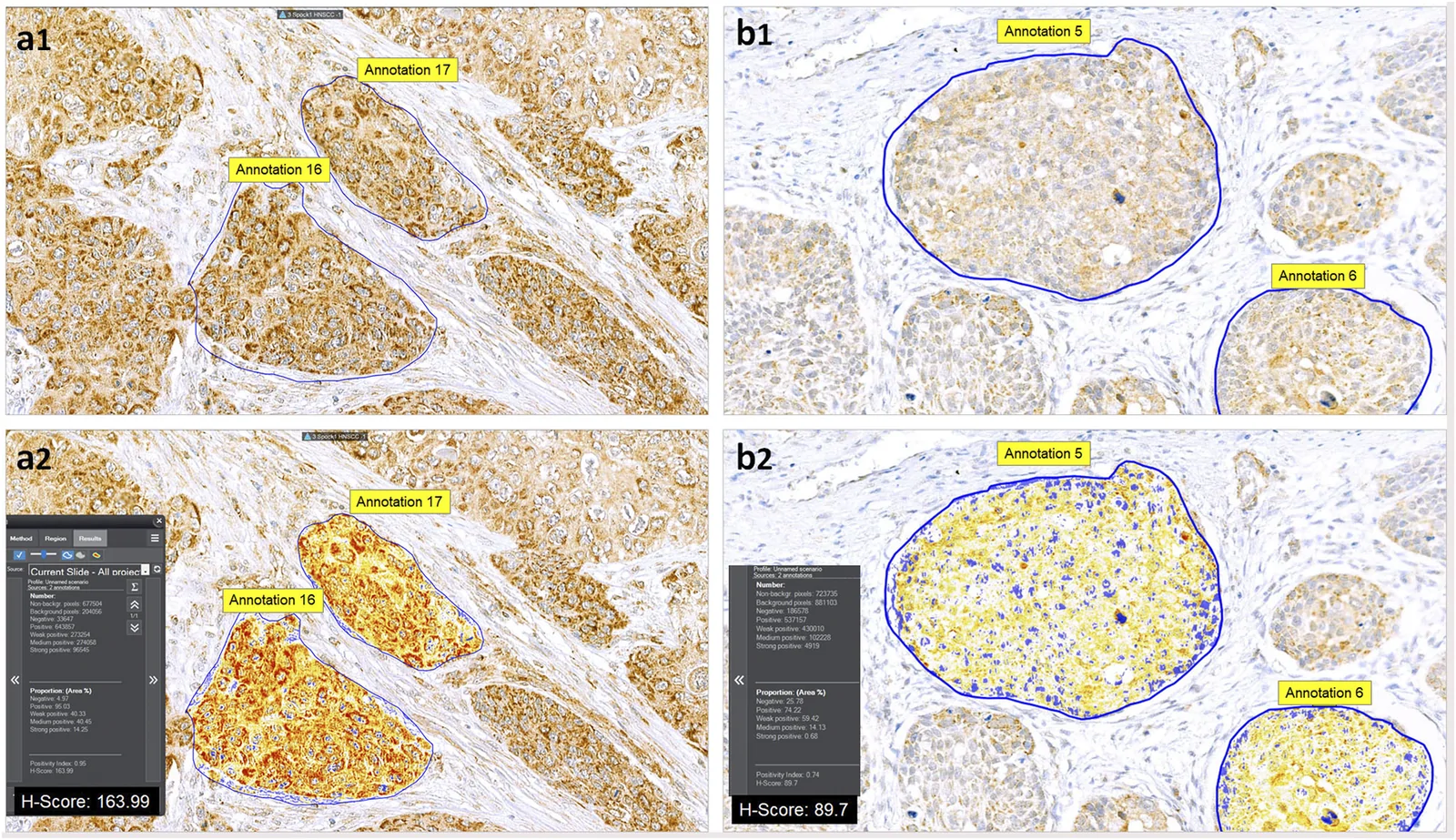
Examples of H-Score values obtained using the DensitoQuant image segmentation algorithm of the QuantCenter program package when evaluating SPOCK1 expression on representative annotations of immunostained digital slides of HNSCC. **(a1,a2)** as well as,** (b1,b2)** are the same areas, whereas **(a2,b2)** show image segmentation results of 163.99 and 89.7 H-Scores, respectively. Intensity categories are shown in blue (0, cell nuclei), lemon yellow (1 - low), orange (2- medium) and red (3 - strong) on A2 and B2. Scale bar: 50 µm.

To complement manual scoring and reduce the risk of bias, SPOCK1 immunostaining was also quantified using the DensitoQuant algorithm of the QuantCenter software package on the same annotations as those tested using eye-control scoring (Figure 4). The same threshold was set up and used for all cases and annotations to ensure a valid comparison of protein expression.

**FIGURE 5:**
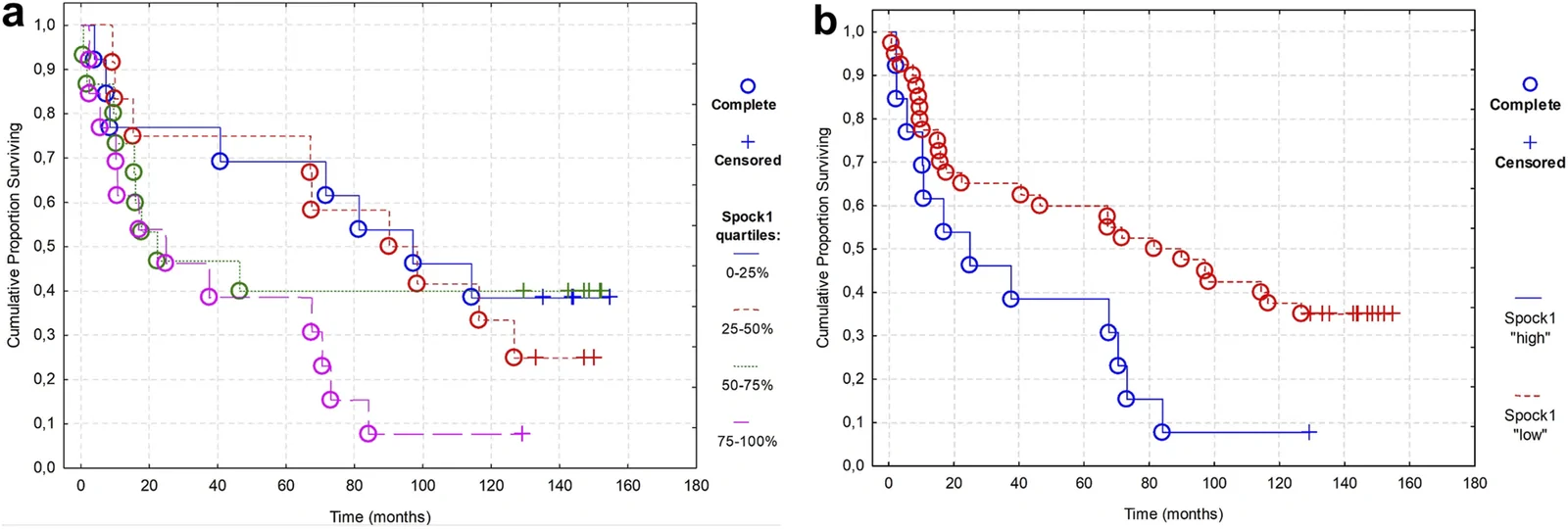
Overall survival (OS) of HNSCC patients based on SPOCK1 immunohistochemistry using H-Score results gained with the DensitoQuant algorithm. Cox-regression analysis of H-Score quartiles visually proved the association between elevated SPOCK1 levels and reduced OS **(a)**. Log-rank test demonstrates that patients with elevated SPOCK1 levels had worse OS, when H-Scores are split at the upper quartile [p = 0.044, WW = 5.309, Test statistic = 2.016; **(b)**].

SPOCK1 protein expression using machine-learning–based quantification of H-Scores showed similar results to the eye-control scoring. Cox-regression analysis similarly proved the association between elevated SPOCK1 levels and significantly reduced OS (p = 0.008, χ2 = 6.076, df = 1) with relative risk (RR) of 1.010 (95% CI: 1.003–1.017) (Figure 5a). However, Chi-square test of the H-Score quartiles did not underlie that elevated SPOCK1 protein levels were associated with significantly poorer OS (p = 0.254, *χ* ^*2*^ = 4.069, df = 3). When splitting SPOCK1 expression at the upper quartile, Log-rank test did show significant survival detriment (p = 0.044, WW = 5.309, test statistic = 2.016, Figure 5b). Splitting at the lower quartile and median did not influence the OS significantly (p = 0.266 and p = 0.178 consequently).

The convergence of manual and algorithm-based approaches reinforces that SPOCK1 expression may serve as a clinically relevant negative prognostic biomarker in HNSCC.

## Discussion

In the oral mucosa of transgenic mice, SPOCK1-mediated pathways may contribute to drug-induced gingival overgrowth and EMT [34, 35]. Also, SPOCK1 has been recently found in a basement membrane-related gene signature, including, resulting in reduced survival of HNSCC [40]. These data suggested that SPOCK1 may have a role in the biology of human oral epithelia including their tumors e.g., HNSCC. Here we tested SPOCK1 expression in human clinical HNSCC samples, both at the mRNA and protein level and confirmed its role in the adverse clinical outcome of HNSCC. Our results also validated the pathologist-read based protein expression results by machine-learning automated scoring.

First in the KM-Plotter open-source database management software, we found that SPOCK1 mRNA levels were significantly higher in oral cancers than in the normal oral mucosa. Subsequently, the „Pan-Cancer” algorithm of the same software revealed that among 500 HNSCC patients those with elevated SPOCK1 mRNA expression showed significantly worse OS, when patients were divided into two groups at the median antigen expression. This was even more emphasized at the high vs. low neoantigen levels and, obviously, when the best prognostic cut-off was applied. At the protein level, SPOCK1 showed a characteristic granular, peri-/paranuclear cytoplasmic immunostaining in HNSCC cells, consistent with the localization to the Golgi-region. This was in line with the observation of detecting testican-1 protein along with amyloid-β precursor protein in the frontal and temporal cortex of Alzheimer patients [41]. Patients with higher SPOCK1 expression exhibited significantly worse OS in the visually scored cohort with the Cox-regression test of H-Score (p = 0.002), and similarly, when cases were dichotomized at the upper quartile of H-Score, with the log-rank test (p = 0.002). The machine-learning-based H-Score evaluation also supported this observation both with Cox-regression test (p = 0.008), log-rank test (p = 0.044). Splitting SPOCK1 expression mostly at the upper rather than the lower quartile, made our results even more easily reproducible, because strong reaction can be straightforward to recognise and score. Our results imply that SPOCK1 may carry an adverse prognostic information. However, we have to accept the limitations of our project concerning the moderate number of patients collected only in a single department. Since not all of our patients had unequivocal HPV data, we could not correlate SPOCK1 expression with virus infection. Also, whole section vs. TMA validation to more accurately consider tumor heterogeneity, and validation of automated vs. pathologist-read testing, might also be required. Therefore, further validation of our findings is needed using large, multicenter HNSCC cohorts accompanied with complete HPV, p16, smoking and alcohol exposure data.

SPOCK1 has been primarily considered as an extracellular matrix protein secreted by mesenchymal fibroblasts, cancer-associated stromal cells (CAFs) [42]. However, it was found also in the nervous system as a synaptic component [43], as well as in chondrocytes and endothelial cells [44] and in different type of cancers mainly of epithelial origin, as a prognostic factor of adverse outcome [23, 45]. SPOCK1 has also been found in the bloodstream of septic patients [46] and *in vitro* studies using SKOV3 ovarian cancer cell lines confirmed that it is released into the culture medium to maintain intracellular equilibrium [33]. If this is the case in the human and serum SPOCK1 may indicate malignization, what we have been investigating in several clinical cancers, SPOCK1 blood level could be a promising biomarker in liquid biopsy for tracing of residual tumors. Though some reports link SPOCK1 expression to EMT and one to the thermoregulation of adipocytes [34, 35, 47], the function of SPOCK1 in epithelial cells is largely unknown. SPOCK1 may support tumor cell survival by inhibiting apoptosis and cooperating with TGF-β in promoting EMT [30]. Our group’s recent investigations in mouse and rat models and in clinical samples demonstrated that SPOCK1 (testican-1) is upregulated during hepatocarcinogenesis and in human hepatocellular carcinomas, but without occurring in the extracellular matrix [24, 45].

In the majority of HNSCC the PI3K-AKT pathway is activated, which can promote proliferation, EMT and tumor cell invasion [48]. HNSCC progression has been linked to ongoing dedifferentiation towards a cancer stem cell state accompanied by “partial” or “hybrid” EMT [37], which are responsible for enhanced tumor cell migration and metastatic spreading [49, 50]. This has been underlined by the detection of several stem cell biomarkers, as mentioned also in the introduction, including CD44 [8], ALDH1 [51], CD133, OCT3, OCT4 [52], SOX2, and NANOG [11, 12]. As we are aware, there has been no relevant mechanism of action data on the potential role of SPOCK1 in HNSCC, we can only speculate based on its relevant pathways and data gained in other malignant tumors. The development of an invasive EMT-prone phenotype can probably be mediated through the activation of TGF-β1, EGFR and NOTCH signaling, as well as hypoxia (HIF-1α) [53]. Indeed, the vast majority of HNSCC overexpress EGFR and can be treated using anti-EGFR antibodies and/or tyrosine kinase inhibitors, which however, frequently become resistant due to EGFR mutation [54]. SPOCK1 expression has also been linked also by several studies with the support of EMT phenotype [36, 37]. Further evidence on the negative prognostic role of SPOCK1 came from a study showing that pre-miR-150 can downregulate SPOCK1 to reduce the aggressiveness of HNSCC cells and colon cancer organoids [55, 56]. These findings are in line with ours, showing that elevated SPOCK1 expression may also be a promoter of the advanced HNSCC, may be EMT-prone phenotype with poor prognosis.

While HPV-positive HNSCC can be prevented by vaccination, HPV-negative cases, particularly at advanced stage, require multimodal radio-/chemotherapy besides surgery [1, 5]. Recently, epidermal growth factor receptor (EGFR)-positive cases have been treated using radiotherapy combined with anti-EGFR chimera (mouse/human) antibodies (cetuximab) [46]. Immune checkpoint inhibitors (e.g., pembrolizumab, or nivolumab) have also been approved in cisplatin-refractory recurrent or metastatic HNSCC [30, 47]. Besides its potential prognostic role, SPOCK1 may be offered as a potential target for developing novel treatment options too.

Taken together, these findings support the concept that SPOCK1 can be a surrogate negative prognostic biomarker in HNSCC. Given its reported involvement in supporting an EMT-related tumor progression pathway, SPOCK1 overexpression may indicate a group of oral cancers with aggressive tumor biology. However, our results may require further validation in larger, independent cohorts, ideally stratified by tumor site, HPV/p16 status, and treatment modalities. If reliably detected in the serum, SPOCK1 may represent a promising target for future biomarker-driven, liquid-biopsy-based monitoring of residual tumors. However, further validation and functional experiments are required to prove our pilot findings.

## Data Availability

The raw data supporting the conclusions of this article will be made available by the authors, without undue reservation.
